# Test-retest reliability of the Mini Nutritional Assessment and its relationship with quality of life in patients with stroke

**DOI:** 10.1371/journal.pone.0218749

**Published:** 2019-06-20

**Authors:** Shu-Chi Lin, Kuan-Hung Lin, Ya-Chen Lee, Hsiao-Yun Peng, En-Chi Chiu

**Affiliations:** 1 Department of Nutrition, Taiwan Adventist Hospital, Taipei, Taiwan; 2 Department of Neurology, Taiwan Adventist Hospital, Taipei, Taiwan; 3 Department of Occupational Therapy, College of Medical and Health Science, Asia University, Taichung, Taiwan; 4 Department of Long-Term Care, National Taipei University of Nursing and Health Sciences, Taipei, Taiwan; Taipei Veterans General Hospital, TAIWAN

## Abstract

**Background & objective:**

Malnutrition is one of commonly issues in patients with stroke. The Mini Nutritional Assessment (MNA) is a widely used measure for assessing nutritional status in patients with stroke. A nutritional measure with acceptable test-retest reliability allows clinicians to consistently assess patients’ nutritional status. Knowledge of the relationship between nutritional status and quality of life (QOL) could guide clinicians to improve QOL in patients with stroke more effectively. This study aimed to examine test-retest reliability of the MNA and its relationship with QOL in patients with stroke.

**Methods:**

Fifty-nine patients participated in the test-retest reliability study and the correlation between the MNA and WHO Quality of Life-BREF (WHOQOL-BREF) study. A repeated-assessments design (1 week apart) was used to examine the test-retest reliability of the MNA.

**Results:**

The intraclass correlation coefficient for the MNA was 0.91. The minimal detectable change and percentage of minimal detectable change for the MNA were 2.1 and 8.2%, respectively. The MNA was positively associated with the QOL (*r* = 0.32; *p* = 0.013). The result of linear regression analysis shows that after controlling for age, sex and activities of daily living functions, only the MNA was significantly associated with the WHOQOL-BREF (*r*^*2*^ = 0.104; *p* = 0.008).

**Conclusions:**

The MNA has satisfactory test-retest reliability that is useful for repeatedly assessing the nutritional status of patients with stroke. The MDC of the MNA has acceptable random measurement error which is useful for determining whether the change score of a patient is outside the range of random measurement error. Future studies that recruit stroke patients in the acute stage is needed to further examine the relationship between the nutritional status and QOL.

## Introduction

Patients with stroke are a highly vulnerable group susceptible to nutritional deficiencies [1]. Malnutrition could have negative effects and affect the ability to perform activities of daily living and may result in poor quality of life (QOL) [2]. Thus, early detection in order to identify those who are in need of nutritional assessment and nutritional care is important.

A variety of assessments have been developed to perform quick screenings for early detection of malnutrition. The Mini Nutritional Assessment (MNA) is one of the most widely used and well known nutritional screening and assessment tools designed for healthy and frail elderly [3, 4]. The MNA has three advantages. First, the MNA is a comprehensive geriatric assessment across four dimensions: anthropometric, global, dietary, and subjective, which covers the areas of cognitive, social, autonomy, and mobility [5, 6]. Second, the MNA does not require any laboratory data. The MNA does not require blood testing to evaluate the nutritional status of subjects, and thus, it is a cost-effective and noninvasive tool. Third, the MNA is quick to administer. It can be completed in less than 15 minutes which improves administrative efficiency and reduces assessment burden on patients and clinicians [5]. Most importantly, the MNA is able to identify people at risk of malnutrition before weight loss occurs. The MNA score for these individuals is between 17 and 23.5 and they are more likely to have decreased caloric intake that can be easily corrected by timely nutritional intervention [5]. The aforementioned advantages of the MNA make it worthy of application in both clinical and research settings.

The MNA has been validated in different populations. The MNA has been validated to be a useful tool in the identification of elderly home-care patients at risk of malnutrition [7]. It is considered a gold standard for free-living elderly [8] and for those living in long-term care facilities [9, 10]. In addition, the MNA has been demonstrated to be feasible and reliable for older people with intellectual disabilities [9]. However, measurement properties tend to be sample-dependent, and thus the evidence established with frail and elderly people cannot be applied to patients with stroke [11].

In addition, to be clinically useful, a measurement tool must be scientifically sound in terms of psychometric properties. Regarding the psychometric properties of the MNA, the correlation between the MNA and the Patient-Generated Subjective Global Assessment was high in screening malnourished elderly patients with stroke [12]. Thus, the MNA appears promising, but the other measurement properties (particularly the test-retest reliability) of the MNA remain unknown for patients with stroke. The test-retest reliability reflects the extent of agreement between repeated measures under similar assessment conditions [13]. A screening tool with acceptable test-retest reliability allows users to consistently identify those at risk for malnutrition [14].

It has been reported that nutritional status was poor in acute stroke patients regardless of level of severity [15] and some studies have reported a relationship between lower nutritional status and lower QOL in patients with cancer and elderly people [16–19]. However, the studies examining the relationship between nutritional status and QOL in patients with stroke are still fewer in numbers. Knowledge of the relationship between nutritional status and QOL could guide clinicians to improve QOL in patients with stroke more effectively. Therefore, the purposes of this study were to investigate the test-retest reliability of the MNA and its relationship with QOL in patients with stroke.

## Methods

### Participants

A convenience sample of outpatients with chronic stroke were recruited from the Department of Health Development at one hospital in northern Taiwan between May 2017 and August 2017. The inclusion criteria for selecting participants were: (1) diagnosis of cerebral hemorrhage or cerebral infarction; (2) having had a stroke for at least 6 months; and (3) the ability to follow verbal instructions to complete the assessment. Participants were excluded if they (1) had unstable medical conditions that may result in re-admission during the study period; (2) were receiving nasogastric tube feeding; and (3) had severe cognitive impairment (the Mini-Mental State Examination, MMSE score of < 17) [20].

The study was approved by the Research Ethics Committee of Taiwan Adventist Hospital (File # 105-E-22). We obtained informed consent for participation from the participants.

### Procedure

A dietitian carried out all assessments, including the MNA and WHO Quality of Life-BREF (WHOQOL-BREF). At the first visit, the dietitian collected demographic information and administered the MNA and WHOQOL-BREF. To investigate the test-retest reliability of the MNA, the MNA was re-administered to the same participants on the seventh day after the first visit.

### Measures

#### MNA

The MNA was developed to identify older adults who have or are at risk for developing malnutrition [3]. The MNA consists of 18 items with a total score ranging from 0 to 30 [21]. A cut-off of ≥ 24 points is regarded as an indication of being well-nourished, 17–23.5 points indicates a risk of malnutrition, and < 17 points indicates the person is malnourished [7]. In the present study, the MNA was assessed using a face-to-face interview format.

#### WHOQOL-BREF

The WHOQOL-BREF was used to evaluate the four subjective quality of life domains: physical, psychological, social relationships, and environment [22, 23]. Each item of the WHOQOL-BREF is scored from 1 to 5, with higher scores indicative of better QOL (score range: 28–140) [22]. In the present study, the Chinese version of the WHOQOL-BREF with 28 items was used and assessed through self-administration. The reliability and validity of the Chinese version of the WHOQOL-BREF have been widely confirmed [23].

#### MMSE

The MMSE was used to examine the cognitive function of participants. It assesses orientation, attention, memory, language, and spatial skills. The total score of the MMSE ranges from 0 to 30, with a score of 24 or greater as no cognitive impairment. In this study, the criterion was set as: no impairment, 24–30; mild cognitive impairment, 18–23; and severe cognitive impairment 0–17 [20].

### Data analysis

#### Test-retest reliability

The intraclass correlation coefficient (ICC_2, 1_) was calculated to examine the extent of agreement between repeated assessments of the MNA. ICC values ≥ 0.80 indicate high agreement, 0.60 to 0.79 indicate moderate agreement, and < 0.59 indicate poor agreement [24].

In addition, the minimal detectable change (MDC) was calculated to estimate the random measurement error of the MNA. The MDC is the smallest threshold of change scores that are beyond random measurement error at a certain level of confidence (usually 95%). The MDC can be used as a threshold to determine whether the change score on a measure of an individual patient has reached a real improvement (or deterioration) [20, 21]. The MDC value was calculated based on the standard error of measurement (SEM) using the following formula [25]:

$$\mathrm{MDC}=z‐{\mathrm{score}}_{\mathrm{level}\;\mathrm{of}\;\mathrm{confidence}}\;x\sqrt{2}\;x\;\mathrm{SEM}$$

$$\mathrm{SEM}={\mathrm{SD}}_{\mathrm{all}\;\mathrm{testing}\;\mathrm{scores}}\;x\;\sqrt{1‐r}$$

In these formulae, the z-score represents the confidence interval from a standard normal distribution (i.e., 1.96 for 95% confidence level in this study). The SD_all testing scores_ means the standard deviation of all scores of the two assessments, and *r* is the ICC value. The multiplier $\sqrt{2}$ indicates the additional uncertainty caused by the use of different scores from two separate assessments [26].

Furthermore, we calculated the MDC% by dividing the MDC by the mean of all scores obtained from the test and retest sessions for both scales and then multiplying by 100. An MDC% less than 30 was considered as acceptable random measurement error [27].

#### Relationship between the MNA and WHOQOL-BREF

Pearson correlation coefficients (*r*) were used to examine the associations between the MNA and the WHOQOL-BREF. An *r* value > 0.75 represents a strong association; values of 0.50 to 0.75, represent moderate association; values of 0.25 to 0.50, indicate mild association; and values ≤ 0.25, indicate weak association [13].

To further examine the relationship between the MNA and the WHOQOL-BREF, a linear regression analysis taking into account age, sex, and activities of daily living functions (measured by the Barthel Index and the Frenchay Activity Index) was conducted. Data were analyzed using SPSS 17.0 for Windows.

## Results

Fifty-nine patients met the selection criteria and participated in the test-retest reliability and the correlation between MNA and WHOQOL-BREF study. The mean age of participants was 71.3 years, and 62.7% of the patients were male. The details of demographic and clinical characteristics of the participants is presented in Table 1. The score of the National Institutes of Health Stroke Scale was estimated from the stroke severity index (called as estimated NIHSS) [28, 29]. The mean score of the MNA in the first assessment was 25.3, indicating that on average, our participants were well-nourished.

**Table 1:** Demographic and clinical characteristics of the participant (N = 59).

Characteristic	
Gender, n (%)	
Male	37 (62.7)
Female	22 (37.3)
Age (years), mean (SD)	71.3 (14.7)
Side of hemiplegia, n (%)	
Right	13 (22.0)
Left	20 (33.9)
Bilateral	26 (44.1)
Time since onset to initial evaluation (months), median (minimum-maximum)	67.0 (12.0–252.0)
Estimated NIHSS, mean (SD)	4.9 (0.3)
Number of stroke occurrences, mean (SD)	1.1 (0.0)
MNA, mean (SD)	
First assessment	25.3 (2.5)
Second assessment	25.8 (2.5)
WHOQOL-BREF, mean (SD)	97.7 (12.8)
MMSE, mean (SD)	28.0 (1.7)

SD: standard deviation; MNA: Mini Nutritional Assessment; WHOQOL-BREF: WHO Quality of Life-BREF; MMSE: Mini-Mental State Examination.

### Test-retest reliability

The ICC for the MNA was 0.91 with 95% confidence of interval of 0.85–8.94. The MDC and MDC% for the MNA were 2.1 and 8.2%, respectively.

### Relationship between MNA and WHOQOL-BREF

The result of Pearson correlation coefficient (*r*) showed that the MNA was positively associated with the QOL (*r* = 0.32; *p* = 0.013). The result of linear regression analysis showed that after controlling for age, sex and activities of daily living functions, only the MNA was significantly associated with the WHOQOL-BREF (*r*^*2*^ = 0.104; *p* = 0.008).

## Discussion

Establishing test-retest reliability of the MNA and its relationship with QOL is important for ensuring the utility of the MNA in patients with stroke. Our findings provide empirical evidence on these important properties of the MNA in patients with stroke for clinicians and researchers.

The results showed that the ICC value of the MNA was 0.91, indicating excellent test-retest agreement. The high ICC value could be attributed to the clear instructions for scoring and sufficient rater training; thus, little variation was found between the scores of each item given by the rater. In addition, the mode of administration, that is, the face-to-face interview method, might have also contributed to the high ICC value in our study. Any unclear meanings could be clarified immediately by our rater during the face-to-face interview. It was reported by our rater that the item “Do they view themselves as having nutritional problems” was easily misunderstood by our participants. The participants had interpreted this question as “Do they have any nutritional related questions”. Both the rigorous rater training and face-to-face interview format indicate that the MNA is particularly useful for repeatedly assessing the nutritional status of patients with stroke.

We found that the MDC values of the MNA was 2.1 points which indicates that a change of more than 2.1 points in the total score is not likely attributed to random measurement error of the measure. The value of MDC provides a threshold for clinicians to determine whether an individual patient has reached a real improvement. Therefore, clinicians can use the MDC value of the MNA to interpret the change in nutritional status of an individual patient after intervention, and develop further treatment plans accordingly. In addition, our results showed that the MDC% (8.2%) of the MNA was below 30% of the mean of all scores of the test-retest assessments, indicating acceptable random measurement error. Thus, the MNA is reliable for screening the nutritional status of patients with chronic stroke.

We found that the MNA was positively associated with the WHOQOL-BREF. Our regression results further support the finding of a positive relationship between nutritional status and quality of life. This finding indicates that the more well-nourished the patients with stroke were, the better their quality of life was. Thus, promoting nutritional status in patients with stroke is important, for it could increase their quality of life. It is worth mentioning that this finding which examines the relationship between the MNA and WHOQOL-BREF, was based on chronic stroke patients (median 67 months after onset). It is believed that patients with stroke in the acute condition may have a higher risk of malnutrition, which could impose considerable impact on QOL [16]. In the future, recruitment of acute stroke patients is needed to further examine the relationship between nutritional status and QOL.

There are many changes in physiological function, nutritional status and diet after stroke. It is important to select a reliable tool to evaluate those patients with stroke who would benefit from early detection of malnutrition [30]. The MNA was developed to identify the risk of malnutrition so as to permit early nutritional intervention when needed. The MNA is not only simple, quick, and easy to use but has also been demonstrated to have excellent test-retest reliability, supporting its utility in clinical and research settings.

### Study limitation

This study has three limitations that should be addressed. First, our sample for the test-retest reliability study was a convenience sample of outpatients with chronic and ischemic stroke. In addition, we excluded patients with severe cognitive impairment and thus all of our participants could report their self-perception of health and nutrition. Therefore, the results of this study may not be generalized to patients in other stages and types of stroke (e.g., acute stage and hemorrhagic stroke) or those with cognitive impairment. Second, we did not obtain the functional prognosis (e.g., the score of the modified Rankin Scale) of our participants, which might have narrowed the interpretation of our findings. Third, most of the participants in this study were well-nourished. To further validate our findings, future studies that recruit stroke patients with diverse of nutritional status are needed.

## Conclusions

Our results showed that the MNA had satisfactory test-retest reliability, indicating that the MNA is reliable in repeatedly assessing the nutritional status of patients with stroke. The MDC of the MNA has acceptable random measurement error which is useful for determining whether the change score of a patient is outside the range of random measurement error. Improving the nutritional status of patients with stroke would be beneficial because nutritional status is significantly correlated with QOL. Our findings provide useful information that could help clinicians in the process of planning nutritional interventions.

## Supporting information

S1 Dataset Raw data of the study. (XLS)
